# Comparing the impact of online and in-person active learning in preclinical medical education

**DOI:** 10.1186/s12909-025-06846-z

**Published:** 2025-03-03

**Authors:** Kiana Malta, Cynthia Glickman, Krystal Hunter, Amanda McBride

**Affiliations:** 1 Cooper Medical School of Rowan University, 401 Broadway, Camden, NJ 08103 USA; 2 Cooper University Hospital, 1 Cooper Plaza, Camden, NJ 08103 USA

**Keywords:** Distance learning, Graduate medical education, COVID-19, Trainees, Virtual

## Abstract

**Background:**

Case-based active learning groups (ALGs) are an approach to learning medicine through the exploration of patient cases in a collaborative small group setting. Owing to the COVID-19 pandemic, this institution had to convert the preclinical curriculum to a virtual format. Currently, few studies have explored the effects of this change on relationships and skills in case-based learning, and our study aimed to explore the impact of the virtual curriculum on future physicians’ interpersonal development and learning preferences.

**Methods:**

An anonymous Qualtrics survey consisting of multiple choice questions (MCQs) and an optional open-ended commentary was distributed to medical students in Classes 2023–2025 enrolled at our institution. Only the MCQs were quantitatively analyzed. Comparisons between 2025 and 2023 and 2024 responses, as well as between 2023 and 2024 responses were performed via chi-square analysis, with an alpha level of 0.05 for all the statistical tests.

**Results:**

A total of 158 responses were collected. The class of 2025 students served as a control, as they enrolled after in-person learning was reinstated, whereas Classes 2023 and 2024 students experienced one year of virtual learning. There was no significant difference between virtual or in-person learning in terms of positive impacts on education (*p* = 0.7), participation (*p* = 0.2), or teamwork (*p* = 0.1), but most students rated these skills overall better with in person delivery methods. Additionally, while there was no significant difference across student groups regarding preference for a delivery method, 50.4% of students preferred a hybrid model versus 40.4% who wanted completely in person format, and a 9.2% minority of students were in favor of fully virtual ALG (*p* = 0.2).

**Conclusions:**

Our study revealed that students do not prefer a completely in-person or virtual format. Our data provide a valuable post pandemic evaluation of preclinical students’ engagement and well-being, suggesting that the skills and relationships fostered through virtual case-based learning are preserved.

## Background

In response to the global novel coronavirus disease-2019 (COVID-19) pandemic, our medical school converted its traditional in-person preclinical curriculum to a completely virtual distance learning format by April 2020. Once COVID-19 cases improved and public health guidelines permitted, the institution switched back to all in-person learning by August 2021. During the early period of the pandemic, education systems around the world faced hurdles such as poor technology infrastructure, social limitations, and health uncertainty that educators and students had to overcome. While it was a radical change for these systems to mobilize quickly, many successful studies, even prior to the pandemic, demonstrated the benefits of virtual distance learning in medical education.

For many years, the ability of medical education to increase educational capacity and equity via simultaneous distance learning has been explored. The interest in technology addressing the shortage of healthcare educators in settings of limited resources, time, and geographic restraints has been present since the internet became more publicly accessible in the 1990s [1]. One of the first challenges was to prove the validity of this format in health education. For example, a cross continental anesthesia education study in 2015 between the United States and Uganda demonstrated large significant increases in post test knowledge acquisition after a series of 24 educational residency lectures provided by videoconferencing [1]. This was a successful example despite some of the challenges with time zone differences and internet access availability.

Additionally, a series of systematic reviews in the literature have attempted to elucidate the value of online vs. offline learning in medical education and have reported positive effects of internet-based interventions [2]; however, either the effect sizes were generally small or were not significantly different from those of offline teaching [3, 4]. One recent systematic review specifically studied medical students due to their common core curricula and institutional exams. The overall findings indicate that online learning works as well as offline learning does, which does not necessarily mean that it works for every student in every context [2]. They hypothesized that online learning would vary from being as effective as offline learning to being more effective depending on the complexity of the learning goals and level of interactivity [2]; thus they proposed a blended learning teaching method for study. On the basis of these findings, Valée et al. hoped to examine specific online intervention effects compared with offline learning. Their systematic review of blended learning (face-to-face with e-learning) analyzed 56 medical education studies from January 1990 - July 2019 and revealed significantly better knowledge outcomes in blended, online, computer-assisted, and virtual patient learning formats than in traditional learning delivery formats [5]. Notably, this study focused on hybrid delivery methods and considered offlineas well as only online single support educational delivery as traditional methods do, making the case for technology to be incorporated as an adjunctive tool in a curriculum. These systematic reviews demonstrate that even before the COVID-19 pandemic, medical education was already open to integrating more online teaching methods and exploring their validity than traditional in-person curricula did. With respect to online case-based learning in health education, in particular, studies have shown that students perform better on multiple choice post tests after a case learning intervention [6] or even on their final semester scores after being entirely online [7]. However, in a scoping review of online case learning, there are variable findings across these studies that also include equivalent or negative findings about the effectiveness of the method and how students or facilitators perceive online case learning [8].

As with any new technology, it comes with its own challenges. Prior to the COVID-19 pandemic, an integrative review revealed four main themes of barriers to the development and implementation of online learning: skills, resources, institutional strategies with support, and attitudes [9]. In an ideal situation, institutions would increase engagement by implementing workshops, providing time to develop online learning resources, and developing infrastructure (of note, lower enrollment online than in person is necessary to reach a break-even point [9, 10]) to improve the collaboration and attitudes surrounding distance learning. However, during the COVID-19 pandemic, many institutions across the country [11] had to quickly convert curricula to virtual learning delivery models without a clear road map for how to make such unprecedented changes.

Medical education is still trying to make sense of the impact of COVID-19 virtual and hybrid learning and how to move forward. There are strengths and weaknesses that must be evaluated to improve and quickly adapt for the future. While virtual teaching offers easier accessibility to learning materials, some have raised concerns about the loss of direct feedback, which could affect future physicians’ skills, such as hands-on anatomy experiences, bedside examinations, and early exposure to clinical skills [12]. For example, in a post COVID-19 survey, US AAMC allopathic anatomy course directors reported a negative impact on the quality of learning due to decreased in-person learning and dissection [13]. Additionally, the pandemic had a different impact on medical students in their preclinical years of training than on senior students at clinical sites. Indeed, prior to the pandemic, the AAMC Y2Q questionnaire for preclinical students indicated that for the year 2019, only 29.4% of respondents said they went “most of the time” to pre-clerkship courses or lectures at their medical schools, and after the pandemic in 2022 there was only a 1% increase in in-person attendance [14, 15]. This highlights the importance of having prepared medical institutions with educational material that supports graduate medical students who are seeking learning models that are blended with both virtual and in person elements [16]. Despite the growing positive perception of online learning materials in the post COVID-19 world, there are persisting barriers to incorporating more virtual learning resources into the curriculum, such as poor institutional support, a lack of dedicated time to create materials, and the misuse of materials, which must be addressed to ensure successful implementation [17].

During the pandemic, lectures and in-person small groups were substituted with a mix of asynchronous pre recorded lectures and synchronous video conferencing sessions respectively. At this institution, preclinical students spend approximately one third of their education in active learning groups, consisting of open discussions of clinical cases to mimic hospital teams evaluating different patient diagnoses. Each group consists of approximately 10 students and 2 facilitators, one clinician and one basic scientist, who help guide students through a learning case over the course of a week. Owing to the timing of the closure of in-person learning delivery and eventual reopening, two classes of the school body, Classes 2023 and 2024, experienced approximately one year of in-person traditional preclinical curriculum and one year of synchronous virtual distance learning for ALG.

### Objective

A majority of studies specific to COVID-19 curriculum changes have examined cross-sectional data from preclinical medical students at the height of the pandemic. These studies examined variables such as the number of hours spent online [18], the perception of online teaching [18], burnout [16, 19], ease of use [20], and satisfaction [21]. However, there has not been a retrospective comparison of virtual and in-person methods of education delivery for case-based learning that evaluate student perceptions of faculty and peer relationships, time management, participation or preferences. Given that two preclinical student cohorts experienced each type of learning method during such a unique time in medical education, it is worth exploring the impact of a virtual curriculum and how future physicians prefer to learn. We hypothesized that these students would prefer the in-person ALG delivery format over the virtual ALG format.

## Methods

### Definitions

When examining the medical education literature, a few terms are used interchangeably and have been implemented in this paper. For the purposes of our study, the terms online learning, virtual learning, and distance learning all refer to the delivery of the curriculum to students at home via the internet during the COVID-19 restrictions. Conversely, traditional learning, offline learning, and in-person learning involve the provision of teaching materials and group discussions face to face with lifted COVID-19 restrictions akin to the pre pandemic delivery format. Finally, blended learning and hybrid learning should be assumed to represent a combination of the elements of distance learning and face-to-face delivery methods.

### Data gathering standards

We conducted and gathered our data for this retrospective study according to the Rowan University research compliance guidelines and received Institutional Review Board approval and exemption IRB#: *PRO-2022-130*. Students provided an electronic informed consent prior to responding to the anonymous Qualtrics© [22] survey. There was no assessed risk to participate in this study and participants did not receive compensation.

### Eligibility criteria

Cooper Medical School of Rowan University medical students enrolled in the classes of 2023, 2024, and 2025 were eligible to participate in this study. We did not poll students graduating after those years, as few students might have experienced both the COVID-19 curriculum and taken multiple years away from school thus joining a later graduation year. No students were excluded on the basis of categorical characteristics such as demographics, or biomedical, or behavioral status.

### Data extraction and synthesis

We determined that the ideal sample size would be 151 participants [23] for our population size, with a confidence level of 90%, and a margin of error of 5%. The Qualtrics© [22] questionnaire consisted of multiple-choice questions (MCQs) and an optional open-ended section if students wanted to comment on their experiences in each of the learning delivery formats. In addition to specific questions about virtual versus in-person ALG, the survey asked the students to indicate the portion of time spent with each ALG delivery method during their preclinical years, their preference for the ALG delivery method, and the influence of facilitators and group members on delivery method preference. Students in the eligible classes were emailed on a weekly basis during the data collection period, and QR codes were accessible in the common areas of the academic building and student lounge to elicit participation. For the ALG opinion MCQs, respondents could indicate “completely agree”, “somewhat agree”, “completely disagree”, “somewhat disagree”, and “not applicable”. During the quantitative analysis, the answers were combined into “agree”, “disagree”, and “not applicable”, and the percentages of student responses from the classes of 2023 and 2024 were calculated. These survey questions were categorized by topics of education, challenges, interpersonal skills, and time management. For the open ended section question, the responses were analyzed qualitatively, and the students’ responses were manually coded. While all the responses were reviewed, not all were included in the final analysis. The reasons for exclusion were that they were not applicable, did not experience virtual ALG delivery methods, or were not written with professionalism. Given the review of an open-ended prompt with a limited sample, performing an inductively coded thematic analysis was more appropriate. Each response was reviewed and coded at the same time for commonalities. Four key themes emerged with analysis: virtual format issues, work life balance, influential group dynamics, and improved connection in-person. The responses included in the results section were edited for readability.

### Statistical methods

The class of 2025 students served as a control for this study, as they enrolled during post pandemic changes and did not experience a year of distance learning. In comparison, the classes of 2023 and 2024 students experienced one year of virtual ALG in either their second or first year of medical school respectively. Chi square comparisons of classes of 2025 with 2023 and 2024 responses as well as 2023 versus 2024 responses were performed. The responses were combined into two categories, “agree” and “disagree”, and the MCQs were analyzed via chi-squared analysis, with an alpha level of 0.05. The chi square method of data analysis was chosen because the survey responses were categorical data, and this study compared the distribution of responses across different medical school cohorts. This approach is suitable because each category is mutually exclusive and the study groups are independent. However, to note some limitations, the sample is a convenience sample as the student populations were surveyed, and the study data were derived from those who responded to the questionnaire only. This method was chosen over randomly selecting students to prioritize an appropriate sample size for the study. Responses to questionnaires by medical students can be limited as they already have many requests for evaluations of courses, facilitators, and other projects.

### Sources of bias

Students were polled regarding their experiences with ALG one year after their return to a traditional curriculum and therefore could experience recall bias they responded. There is also voluntary response bias as the sample consists of self-selected volunteers. Additionally, the Class of 2025 experienced two sessions of ALG virtually in January 2023 due to an unforeseen COVID-19 cluster outbreak at the school and thus did have ALG virtually, but not significantly enough to contribute to their experience with the distance learning format. Their responses could be biased from those sessions.

## Results

### Population characteristics

The study received 158 responses from a total of 341 eligible students from classes of 2023–2025, consisting of 46 from 2023, 75 from 2024, and 37 from 2025, for a 46.3% total response rate. A majority of respondents from Classes 2023 and 2024 (94.7% and 95.8% respectively) indicated that they spent at least 50% of their preclinical ALG education virtually, meaning that at least one year of their preclinical education was virtual.

### Comparative analysis

We investigated the potential differences in response between the students who had at least half their preclinical medical education through virtual learning, i.e., the classes of 2023 and 2024, and those who had none, i.e., like the class of 2025. When performing Chi-squared analysis of the responses for classes of 2023 and 2024 with the Class of 2025, there were no significant differences in any of the responses. Given that each class experienced distance learning in either their first year or second year of medical school, we compared the class of 2023 with the class of 2024 survey responses to determine whether the order in which virtual and traditional learning occurred mattered. There was no significant difference between the two classes’ responses, with the exception of one survey question: “I felt more confident contributing to ALG when I was in person than in virtual ALG”. Students in the Class of 2024, who started in the virtual ALG format first, were significantly more confident in contributing to ALG than their classmates in the Class of 2023, who started their preclinical years of medical school in person (*p* = 0.04).

### Questionnaire data

Students from the classes of 2023 and 2024 (*n* = 109) were surveyed on their opinions regarding several aspects of their traditional learning format compared with the virtual method of ALG. They provided their perspective regarding the impact of the method on education, challenges, interpersonal skills, and time management (see Table 1). In terms of education and interpersonal skills, a majority of the respondents indicated that in person ALG was better for their education (85.1%), participation (66.9%), camaraderie (90.1%), teamwork (79.3%), confidence (52.9%), and relationships with faculty (86.8%). On the other hand, the students responded that they had a better work-life balance (71.1%) with the virtual learning environment and did not face greater academic challenges (59.5%) when they participated virtually.

**Table 1:** Questionnaire data for students from the classes of both 2023 and 2024 (*n* = 109), who experienced one year each of virtual ALG. Overall, these students indicated more positive experiences with the in-person delivery method of ALG with respect to their education 85.1% (question 1), participation 66.9% (question 2), camaraderie 90.1% (question 8), better relationships with faculty 86.8% (question 9), and teamwork 79.3% (question 10). However, 71.1% of the students did not indicate a better work-life balance during in-person ALG than during virtual ALG (question 12)

Survey Questions	Disagree (%)	Agree (%)	Not Applicable (%)
**Education**			
1. In person ALG more positively impacted my education than virtual ALG	14.0	85.1	0.9
2. I participated more in person at ALG than virtual ALG due to format delivery	27.3	66.9	5.8
3. I felt more confident contributing to ALG when I was in person than virtual	43.8	52.9	3.3
4. I prepared for ALG cases more with in person than virtual ALG	39.7	52.1	8.2
5. I used ALG materials to study for institutional exams more with in person ALG than virtual ALG	47.9	37.2	14.9
**Challenges**			
6. I faced more academic challenges participating virtually in ALG than in person	59.5	33.0	7.5
7. I faced more personal challenges participating virtually than in person ALG	43.8	47.9	8.3
**Interpersonal Skills**			
8. I experienced more camaraderie with my peers in person ALG than virtual ALG	9.1	90.1	0.8
9. I experienced better relationship with my ALG faculty in person ALG than virtual ALG	10.7	86.8	2.5
10. I experienced better teamwork with my peers in person ALG than virtual ALG	19.8	79.3	0.9
**Time Management**			
11. In person ALG more positively impacted my time management skills than virtual ALG	45.5	51.2	3.3
12. I had a better work life balance during in person ALG than virtual ALG	71.1	23.1	5.8

### Student preferences

**Fig. 1:**
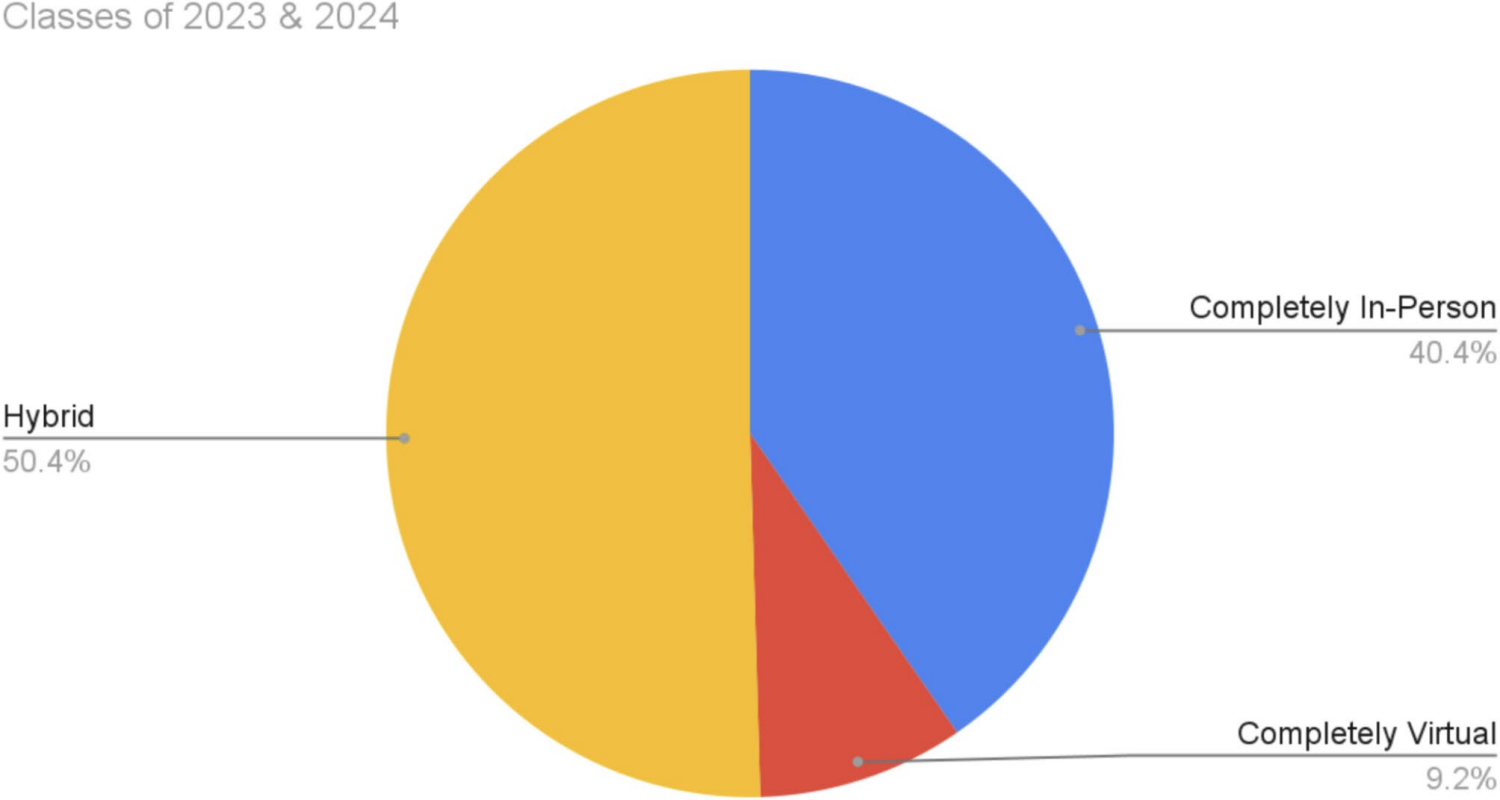
Students preferences for ALG delivery method: Class of 2023 and 2024 preferences for ALG between 100% in-person, 100% virtual, and a hybrid course option. This finding indicates that 50.4% of the respondents preferred a hybrid option for ALG whereas 40.4% said that they would rather have an entirely in-person ALG class. The least popular option for ALG was the completely virtual option, with only 9.2% of the students being in favor

**Fig. 2:**
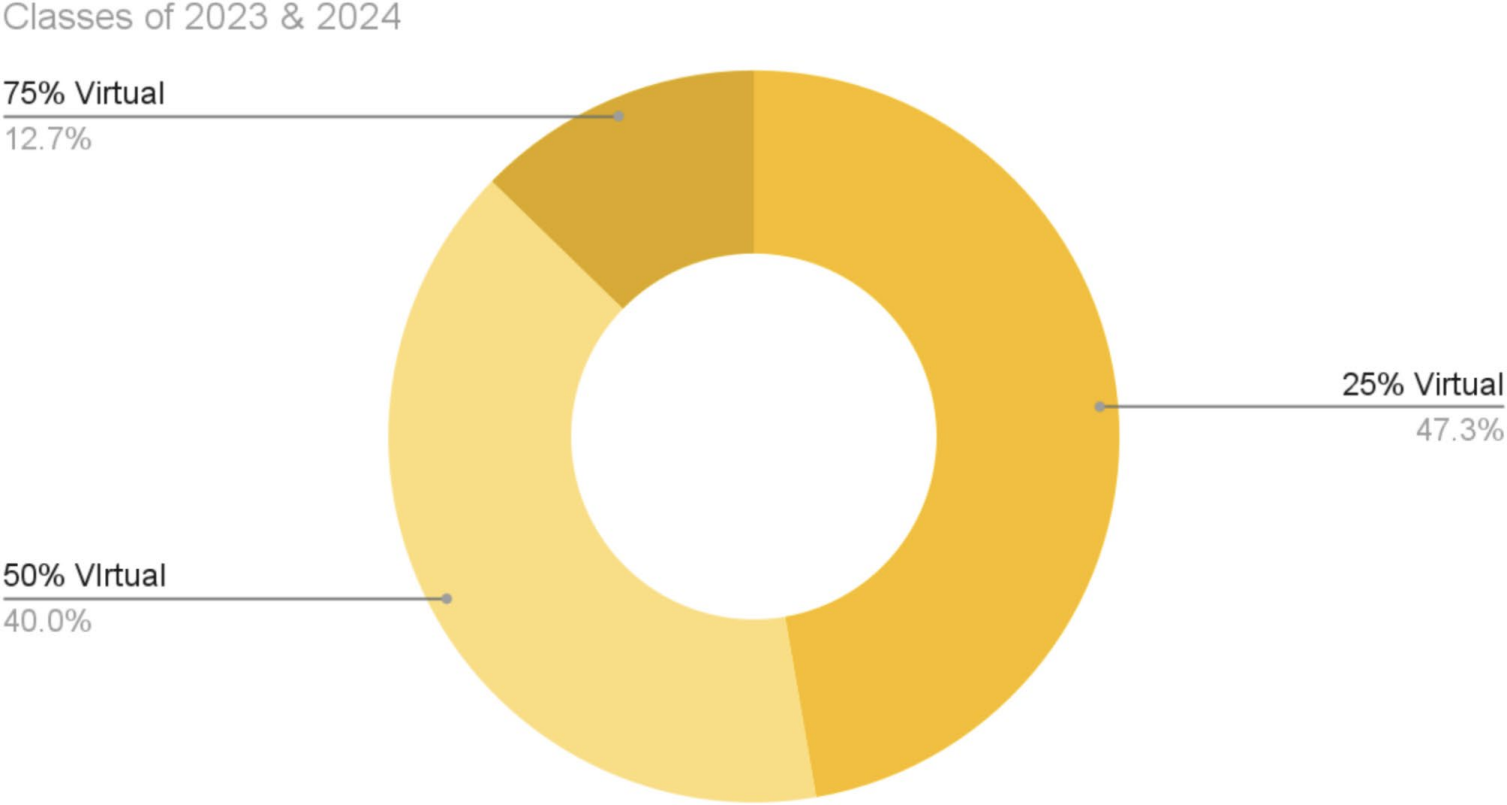
Hybrid preferences: Among the students from the classes of 2023 and 2024 who indicated their preference for a hybrid ALG option (*n* = 55), 47.3% wanted a quarter of the curriculum to be a virtual delivery method. To characterize this further, 40% of the respondents wanted half the ALG curriculum to be virtual, and 12.7% of the students preferred three quarters of ALG sessions to be virtual

The participants indicated their preferences for the ALG delivery method on the questionnaire. Overall, a hybrid option was preferred by 50.4% of the students from the classes of 2023 and 2024, whereas 40.4% of the students indicated a completely in-person ALG was favored. In the minority, only 9.2% of the students indicated a preference for an entirely virtual delivery method for ALG (see Fig. 1). The respondents could choose options of 0%, 25%, 50%, 75%, and 100% virtual learning formats for ALG (see Fig. 2). Among the students from classes 2023 and 2024 who chose a hybrid preference (*n* = 55), 47.3% chose a 25% virtual hybrid ALG and 40.0% chose a 50% virtual hybrid ALG delivery format. The classes of the 2023 and 2024 students were also asked to indicate their opinions as to whether their relationships with their ALG facilitators and group members influenced how they perceived the virtual or in-person format as preferable. For this question, 21.7% and 25.3% of respondents from the classes of 2023 and 2024 respectively agreed that there was facilitator and group influence on their preferred ALG delivery method.

### Qualitative analysis

At the end of the survey, the students were given the opportunity to share their opinions and perspectives in an open-ended prompt (see Table 2) about the virtual and in-person delivery formats. Four key themes were detected through inductive coding of student responses including virtual format issues, work-life balance, influential group dynamics, and improved connections in-person. Some students indicated technical issues, poor engagement and attention with the virtual format ALG delivery. Despite those issues, other students endorsed a preference for virtual or hybrid options for ALG, as it gave them improved schedule flexibility. They also reported that their experiences and preferences for ALG were heavily influenced by their facilitators and group members, regardless of delivery method. Additionally, the students indicated a better learning experience in person because of their ability to work and engage with their peers face to face.

**Table 2:** Open ended survey question that students from the classes 2023 and 2024 completed with quotes about their experiences with the virtual and in person ALG formats. Overall, the students indicated that they preferred a more peer-connected learning environment, but hybrid/virtual learning was better for their work-life balance. They also reported that their experience, regardless of delivery method, was heavily influenced by the milieu of their group members and facilitators. Finally, there were technical issues, poor engagement and reduced attention that accompanied the virtual format delivery method

Open Ended Survey Responses
**Virtual Format Issues**
*“Virtual made it easier for students to disengage*,* which made it more frustrating. ALG would be nice if it was in person Monday/Wednesday and virtual for wrap up on Fridays. This would force everyone to be in person for the majority of the discussions*,* and then on Friday allow groups to finish up in a timely manner”*
*“I retained very little information discussed in ALG virtually compared to in person ALG”*
*“I generally felt it was easier to not participate during virtual ALG sessions compared to in person sessions. It was easier not to prepare as well and let others lead the discussion”*
*“Technical problems and poor audio quality impeded my learning when doing virtual ALG”*
**Work Life Balance**
*“Virtual ALG is way better than in-person*,* it should be twice weekly”*
*“Virtual was very nice during exam weeks or Fridays because I could work at home without transit time and it allowed me to see friends and family on weekends while still tuning into class”*
*“I cannot comment on the work life balance of virtual vs in-person ALG because there was a pandemic happening so it would be more affected by that than how my ALG was set up”*
*“I would be much happier if ALG was virtual*,* at least on Fridays. It would give more flexibility which would reduce stress for students. In general*,* I think that we could get the same benefit from ALG if it was only twice a week”*
*“I think in person ALG was better for the ALG learning experience. However*,* virtual ALG was better for work life balance. So*,* I think the best scenario would be a hybrid ALG model*,* with ALG primarily being in person*,* but able to switch to virtual before exams or busy time periods”*
*“Having early AM in person ALG was detrimental to my time management and health. Online ALG saves some commute time and allows for better sleep*,* which in turn raised my grade as I have a hard time falling asleep early”*
**Influential Group Dynamics**
*“The quality of ALG is largely facilitator-dependent”*
*“My facilitators helped make virtual ALG enjoyable*,* but I would rather have had an in person opportunity with them”*
*“My M1 ALG facilitators (virtual) had higher academic expectations and the group had a more lively dynamic*,* which had a larger impact on my learning than the format of delivery”*
*“It is difficult comparing in-person vs virtual ALG because we were in two different groups both years. I meshed way better with the facilitators and group members in my virtual ALG but I still preferred to come in-person”*
*“Having different ALG facilitators between first and second year affected my answers. (Thought M2*,* although virtual*,* was more effective for learning)”*
*“I believe the students and faculty I was placed with during my virtual experience were simply more enjoyable to work with than those during my in person experience*,* I believe this influenced my views significantly”*
**Improved Connection While in Person**
*“Overall better case engagement and flow during in person ALG*,* which translates to better team dynamic and comfortability while on the Floors during the 3rd year”*
*“Students are definitely more personable and in more of a conversational setting when in person”*
*“In person ALG*,* would likely be a good thing for beginning first years who are just getting established and meeting their classmates. More experienced students would likely benefit from ALG transitioning to virtual. Perhaps first years can have in person and ALG switches to virtual for second year students”*
*“I enjoyed the in person experience more because it was nice to be able to interact in person with my peers. It was just different and better in person”*

## Discussion

In our study of the ALG delivery format, we first assessed whether there were any significant differences between each medical student class. When comparing the responses of Class 2025 with those of Class of 2023 and 2024 through chi square analysis, no significant differences were found between the respondents’ data. This demonstrates that regardless of delivery format, the students indicated that their experiences were relatively similar. There was no significant difference between the classes of 2023 and 2024, who experienced both curriculum formats in different preclinical years because of the pandemic. Thus, the order in which students experienced virtual learning ALG did not matter. Only with respect to the question regarding students’ confidence in contributing to ALG, was there a significant difference between the classes of 2023 and 2024 responses. Students who started in a virtual format, the Class of 2024, indicated that they felt more confident while contributing to ALG in-person than those that started in a traditional format, the Class of 2023 (*p* = 0.04). This could be explained by the fact that the Class of 2023 started ALG with an in-person delivery method and could have been less confident in general since that was also the first year of medical school for that cohort.

One can see how students felt about each delivery method after a more specific review of the Class of 2023 and 2024 responses to each of the survey questions detailed in Table 1. For the questions pertaining to education, 85.1% of the students indicated a more positive impact, and 66.9% had greater participation with an in-person delivery method of ALG on Questions 1 and 2 respectively. 90.1% of students also experienced more camaraderie with in-person ALG on Question 8, 86.8% had better relationships with faculty per Question 9, and 79.3% felt that there was better teamwork per Question 10 with respect to interpersonal skills. However, 71.1% of the students did not indicate a better work-life balance during in person ALG than during virtual ALG on Question 12 in the area of time management. The data from our survey generally show that students prefer an in-person ALG delivery format because of its positive impact on education and ability to form better relationships, but do not find it beneficial for work-life balance, suggesting that a hybrid method of ALG delivery could be a potential solution to retain the best elements of each option. Indeed, when their preference for an ALG delivery method was queried, there was no significant difference between respondents in terms of their preference for the delivery method. Among the classes of 2023 and 2024, 50.4% of the students indicated their choice of hybrid compared with 40.4% who desired completely in person and a small minority of students, 9.2%, who wished for a completely virtual option. After reviewing the open-ended question qualitative results in Table 2, one can gain a better understanding of the nuances from the experience of virtual versus in person that make sense of more students still indicating a preference for a hybrid delivery method. Some of the students’ responses that illuminate the troubles with a completely virtual format highlight attention difficulty, poor retention of information, decreased participation, and technical difficulties while in the online format. Indeed, perhaps the distance of a virtual format offered an illusion of not having to engage as fully and falling short of peer and/or facilitator expectations to participate more equally. Students indicated improved connection and engagement with peers with in-person delivery, but virtual ALG provided students with increased flexibility in their schedule, which they commented allowed them more support from friends and family and reduced stress as they had more autonomy to delegate how their time was spent. Interestingly, during the coding process for thematic analysis the theme of influential group dynamics was created as multiple students indicated that the delivery method was secondary to who was part of their ALG group. For both the virtual delivery method and the in-person delivery method, the students posited that their specific facilitators and peers had a greater impact on their learning than did the actual ALG method. This further illustrates the benefit of a hybrid delivery method as students could use the time in person to form better connections with their group while maintaining a positive learning environment with better work-life balance during virtual sessions.

Given the findings of this study, we believe that students do not widely prefer a completely in-person or virtual format and could benefit from a blended ALG curriculum. Indeed, in the present state of the medical world, which increasingly uses electronic-based healthcare delivery, having medical students who are more facile with technology could improve their ability to adapt. More broadly, several recent studies agree with our findings within the field of graduate medical education. A U.S. dental school reported that 80% of their students wished to continue some virtual education after the pandemic and that their virtual cohort was either as likely or more likely to receive high scores than their face-to-face counterparts in 16/17 courses [24]. Additionally, an anatomy lab in Genoa, Italy, delivered 160 h of virtual learning during the pandemic in their anatomy course and reported that students scored higher in virtual teaching than in person teaching [25]. However, this comes with challenges in restructuring medical education such that the integrity of in-person skills that come from working with peers and facilitators is not lost. In a position paper, a Family Medicine Group from Cairo called for global action to assess how to prepare a medical curriculum in the post-COVID-19 world [26]. A key to this will be identifying each of the stakeholders in the process. Institutions have been working on assessing the needs of their learners and preparing faculty to meet these new expectations. Some choose to create workshops, individual support, and as needed support for their students and faculty in low-pressure settings to transition adult learners to distance learning formats [27]. This issue also significantly impacts trainees and faculty who are working in the clinical setting and who complete didactics as part of their training. In one study, for didactics that are lecture based or presentation based, learners preferred a distance format, whereas small groups or training sessions were better suited for in-person delivery. Ultimately, a hybrid approach was preferred by staff and trainees for the most advantageous learning environment [28]. For courses or educational sessions that typically require more hands-on learning or clinical skills, we need to look forward to new innovations to modernize the medical school curriculum. There are already interventions that have started to incorporate new technologies such as artificial intelligence with real cases and language processing technology to create virtual case learning environments for students [29]. Future studies should focus on the implementation of these new virtual tools in the medical curriculum where applicable to prepare future physicians for a reality where they will be part of the workplace.

### Limitations

To properly weigh this study’s findings, its boundaries must also be discussed. First, this study sent a survey to a small sample of medical students at a single institution in the northeastern USA. The generalizability of our findings could be limited, for example, compared to other institutions with variable class sizes in different geographic locations. It must be considered that other institutions have entirely different resources, infrastructures, and staff training to implement online vs. in-person curricula, which may not have posed the same challenges. The data are also collected from students who opted to complete the survey; therefore, they are subject to nonresponse bias. Some ways the team tried to reduce this were by keeping the survey short, with various distribution methods (e-mail, QR codes displayed in school common areas), and keeping the survey anonymous. Further investigation of the experiences of medical students who were exposed to hybrid or online curricula during the COVID-19 pandemic is needed. Additionally, for the purpose of this study, only active learning groups were the focus of the study. Future research investigating the impact of delivery methods on different courses in medical education is needed.

## Conclusions

Our post-pandemic evaluation of preclinical ALGs indicates that students prefer the traditional model for interpersonal learning but would like to retain distance learning for work-life purposes. Based on the current literature, it is worth noting that a blended learning environment would make the best of both formats to the advantage of students’ learning.

## Data Availability

The data sets generated and/or analyzed during the current study can be found in the Figshare repository, DOI: 10.6084/m9.figshare.25321012.

## Abbreviations

ALG: Active Learning Group

## References

[1] Kiwanuka JK, Ttendo SS, Eromo E, Joseph SE, Duan ME, Haastrup AA et al. Synchronous distance anesthesia education by internet videoconference between Uganda and the United States. J Clin Anesth. 2015;27(6):499–503. 10.1016/j.jclinane.2015.04.004 26001319

[2] Pei L, Wu H. Does online learning work better than offline learning in undergraduate medical education? A systematic review and meta-analysis. Med Educ Online. 2019;24(1):1666538. 31526248 10.1080/10872981.2019.1666538 PMC6758693

[3] Cook DA, Levinson AJ, Garside S, Dupras DM, Erwin PJ, Montori VM. Internet-based learning in the health professions: a meta-analysis. JAMA. 2008;300(10):1181–96. 18780847 10.1001/jama.300.10.1181

[4] Richmond H, Copsey B, Hall AM, Davies D, Lamb SE. A systematic review and meta-analysis of online versus alternative methods for training licensed health care professionals to deliver clinical interventions. BMC Med Educ. 2017;17(1):227. 29169393 10.1186/s12909-017-1047-4 PMC5701457

[5] Vallée A, Blacher J, Cariou A, Sorbets E. Blended learning compared to traditional learning in medical education: systematic review and meta-analysis. J Med Internet Res. 2020;22(8):e16504. 32773378 10.2196/16504 PMC7445617

[6] Vedi N, Dulloo P. Students’ perception and learning on case based teaching in anatomy and physiology: an e-learning approach. J Adv Med Educ Prof. 2021;9(1):8–17. 33521136 10.30476/jamp.2020.87332.1304 PMC7846716

[7] Leavy JE, Della Bona M, Nelson B, Leaversuch F. A comparison of face-to-face and fully online problem-based learning: student results and staff experiences, 2014–2020. Health Promot J Aust off J Aust Assoc Health Promot Prof. 2022;33(Suppl 1):57–66. 10.1002/hpja.636 PMC9796957 35856188

[8] Donkin R, Yule H, Fyfe T. Online case-based learning in medical education: a scoping review. BMC Med Educ. 2023;23(1):564. 37559108 10.1186/s12909-023-04520-w PMC10413534

[9] O’Doherty D, Dromey M, Lougheed J, Hannigan A, Last J, McGrath D. Barriers and solutions to online learning in medical education - an integrative review. BMC Med Educ. 2018;18(1):130. 29880045 10.1186/s12909-018-1240-0 PMC5992716

[10] Maloney S, Nicklen P, Rivers G, Foo J, Ooi YY, Reeves S, et al. A cost-effectiveness analysis of blended versus face-to-face delivery of evidence-based medicine to medical students. J Med Internet Res. 2015;17(7):e182. 26197801 10.2196/jmir.4346 PMC4527010

[11] Hilburg R, Patel N, Ambruso S, Biewald MA, Farouk SS. Medical education during the Coronavirus disease-2019 pandemic: learning from a distance. Adv Chronic Kidney Dis. 2020;27(5):412–7. 33308507 10.1053/j.ackd.2020.05.017 PMC7309716

[12] Park A, Awan OA. COVID-19 and virtual medical student education. Acad Radiol. 2023;30(4):773–5. 35667980 10.1016/j.acra.2022.04.011 PMC9021358

[13] Shin M, Prasad A, Sabo G, Macnow ASR, Sheth NP, Cross MB, et al. Anatomy education in US medical schools: before, during, and beyond COVID-19. BMC Med Educ. 2022;22(1):103. 35172819 10.1186/s12909-022-03177-1 PMC8851737

[14] AAMC Medical School Year Two Questionnaire. Available from: https://www.aamc.org/media/43656/download

[15] AAMC 2022 Y2Q All Schools Summary Report. Available from: https://www.aamc.org/media/66071/download#:~:text=The total share of Y2Q,seven points lower (34.2%25).

[16] Alrumi N. The impact of COVID-19 on medical education and training. Br J Hosp Med Lond Engl 2005. 2024;85(5):1–7. 10.12968/hmed.2023.0462 38815970

[17] Kim KJ, Kim G, Kang Y. Faculty perceptions and use of e-learning resources for medical education and future predictions. Korean J Med Educ. 2023;35(4):325–34. 38062680 10.3946/kjme.2023.270 PMC10704051

[18] Dost S, Hossain A, Shehab M, Abdelwahed A, Al-Nusair L. Perceptions of medical students towards online teaching during the COVID-19 pandemic: a national cross-sectional survey of 2721 UK medical students. BMJ Open. 2020;10(11):e042378. 10.1136/bmjopen-2020-042378 PMC7646323 33154063

[19] Hunt S, Simpson J, Letwin L, MacLeod B. Is online learning during the COVID-19 pandemic associated with increased burnout in medical learners? A medical school’s experience. PLoS ONE. 2023;18(5):e0285402. 37146035 10.1371/journal.pone.0285402 PMC10162515

[20] Alsoufi A, Alsuyihili A, Msherghi A, Elhadi A, Atiyah H, Ashini A, et al. Impact of the COVID-19 pandemic on medical education: medical students’ knowledge, attitudes, and practices regarding electronic learning. PLoS ONE. 2020;15(11):e0242905. 33237962 10.1371/journal.pone.0242905 PMC7688124

[21] Salih KM, Elnour S, Mohammed N, Alkhushayl AM, Alghamdi AA, Eljack IA, et al. Climate of online e-Learning during COVID-19 pandemic in a Saudi Medical School: students’ perspective. J Med Educ Curric Dev. 2023;10:23821205231173492. 37153851 10.1177/23821205231173492 PMC10159252

[22] Qualtrics USA. Qualtrics; 2024. Available from: https://www.qualtrics.com

[23] Sample size calculator. Qualtrics Sample size calculator. 2023. Available from: https://www.qualtrics.com/blog/calculating-sample-size/

[24] Zheng M, Bender D, Lyon C. Online learning during COVID-19 produced equivalent or better student course performance as compared with pre-pandemic: empirical evidence from a school-wide comparative study. BMC Med Educ. 2021;21(1):495. 34530828 10.1186/s12909-021-02909-z PMC8443899

[25] Zarcone D, Saverino D. Online lessons of human anatomy: experiences during the COVID-19 pandemic. Clin Anat N Y N. 2022;35(1):121–8. 10.1002/ca.23805 PMC9298225 34704281

[26] Andraous F, Amin GEAD, Allam MF. The new normal for medical education during and post-COVID-19. Educ Health Abingdon Engl. 2022;35(2):67–8. 10.4103/efh.efh_412_20 36647934

[27] Samuel A, Teng Y, Soh MY, King B, Cervero RM, Durning SJ. Supporting the transition to distance education during the pandemic and beyond. Mil Med. 2023;188(Suppl 2):75–80. 37201481 10.1093/milmed/usac217

[28] Evans AZ, Adhaduk M, Jabri AR, Ashwath ML. Is virtual learning here to stay? A multispecialty survey of residents, fellows, and Faculty. Curr Probl Cardiol. 2023;48(6):101641. 36773945 10.1016/j.cpcardiol.2023.101641 PMC9911980

[29] Wang M, Sun Z, Jia M, Wang Y, Wang H, Zhu X, et al. Intelligent virtual case learning system based on real medical records and natural language processing. BMC Med Inf Decis Mak. 2022;22(1):60. 10.1186/s12911-022-01797-7 PMC8895690 35246134

